# Platelet Indices in Colorectal Cancer Patients with Synchronous Liver Metastases

**DOI:** 10.1155/2019/6397513

**Published:** 2019-11-04

**Authors:** Li Li, Xiao-Yi Huang, Na Li, Ming-ming Cui, Rui-tao Wang

**Affiliations:** ^1^Department of Colorectal Surgery, Harbin Medical University Cancer Hospital, Harbin Medical University, Harbin, Heilongjiang 150081, China; ^2^Biotherapy Center, Harbin Medical University Cancer Hospital, Harbin Medical University, Harbin, Heilongjiang 150081, China; ^3^Department of Internal Medicine, Harbin Medical University Cancer Hospital, Harbin Medical University, Harbin, Heilongjiang 150081, China

## Abstract

**Aims:**

Liver metastases occur in approximately 25% of colorectal cancer (CRC) patients and cause more than 90% of deaths in CRC. Platelets play a crucial role in cancer progression and metastases. We aimed to investigate the relationship between platelet indices and CRC with synchronous liver metastases.

**Methods:**

We conducted a retrospective clinical study including 206 CRC patients without metastases and 200 CRC patients with synchronous liver metastases from January 1, 2015, to December 31, 2017. Data of the patients' clinicopathological characteristics were collected.

**Results:**

Platelet distribution width (PDW) was decreased in CRC patients with liver metastases compared with CRC patients without liver metastases. In addition, the prevalence of liver metastases reduced as PDW quartiles increased. After adjusting for other risk factors, the odds ratios (95% confidence intervals) for CRC liver metastases according to PDW quartiles were 1.000, 0.289 (0.156-0.535), 0.482 (0.271-0.860), and 0.190 (0.101-0.358).

**Conclusions:**

Compared with CRC patients without metastases, PDW is reduced in CRC patients with liver metastases. Moreover, PDW was independently associated with the presence of CRC liver metastases.

## 1. Introduction

Liver metastases occur in approximately 25% of colorectal cancer (CRC) patients and cause more than 90% of deaths in CRC [1]. CRC patients with liver metastases have an estimated 5-year survival of 38% [2]. Therefore, identification of novel serum biomarkers for CRC with liver metastases is urgently needed.

Platelets act as a crucial role in tumor growth and metastasis [3, 4]. Mean platelet volume (MPV) reflects platelet size and indicates platelet activation in clinical practice [5]. Platelet distribution width (PDW) reflects variation in platelet size and differentiates thrombocytopenia [6]. MPV was found to be altered in numerous tumors, such as lung, breast, gastric, ovarian, and colorectal cancers [7–11]. Our previous study also revealed that elevated MPV predicts a worse prognosis in CRC patients [12]. However, there is no study investigating the clinical implications of platelet indices in metastatic CRC patients.

We aimed to evaluate the relationship between platelet indices and CRC with liver metastases.

## 2. Methods

### 2.1. Study population

From January 2015 to December 2017, 200 CRC patients with synchronous liver metastases and 206 CRC patients without metastases at the Harbin Medical University Cancer Hospital were included in this study. CRC was histologically diagnosed. CRC synchronous liver metastases were defined as liver metastases detected at or before diagnosis of CRC [13]. Liver metastases were assessed with liver magnetic resonance imaging (MRI). All patients received MRI scanning of the head, CT lung screening, liver MRI, and ^99m^Tc-MDP bone scan. The exclusion criteria were the following: (1) underwent radiotherapy or chemotherapy prior to the enrolment, (2) had hematological disorders, (3) administration of acetylic salicylic acid, and (4) had other organ metastasis besides the liver.

The study was approved by the institutional review boards of Harbin Medical University Cancer Hospital. The informed consent was waived because this was a retrospective study.

### 2.2. Statistical Analysis

Student's *t*-test (for continuous variables with normal distribution), Mann–Whitney *U* test (for continuous variables with nonnormal distribution), and *χ*^2^ test (for categorical variables) were used to analyze the differences between two groups. Multivariate logistic regression analysis was used to calculate the odds ratios and 95% confidence intervals for liver metastasis adjusting for other confounding factors. *P* < 0.05 was considered statistically significant. All analyses were performed by using SPSS Statistics version 22.0 (SPSS Inc., Chicago, IL, USA).

## 3. Results

Table 1 summarizes the clinicopathological characteristics of CRC patients. Of the 406 CRC patients enrolled, 236 (58.1%) were men and 170 (41.9%) were women. The patients with liver metastasis had a lower albumin, haemoglobin, and PDW and higher white blood cell counts. The number of patients with poor differentiation was 48 and 56 in the metastasis and nonmetastasis groups, respectively. However, statistical significances were not found in age, gender, smoking status, drinking status, fasting plasma glucose, platelet count, and MPV levels between two groups.

The platelet indices in CRC patients were shown in Table 2 and Table 3. In the group of nonliver metastasis, platelet count was significantly associated with T stage. MPV was associated with tumor size and PDW with lymph node metastasis. In the group with liver metastasis, we failed to observe the associations between platelet indices and primary tumor location, tumor size, differentiation, T classification, lymph node metastasis, metastatic tumor size, and metastatic tumor nodules.

The ROC curve for metastasis was used to verify the optimum cutoff points for PDW. A PDW ≤ 16.5% was shown to be predictive for CRC liver metastasis, with a sensitivity of 51.0% and a specificity of 85.2% (area under the curve 0.712; 95% confidence interval 0.659-0.766; *P* < 0.001).

**Figure 1:**
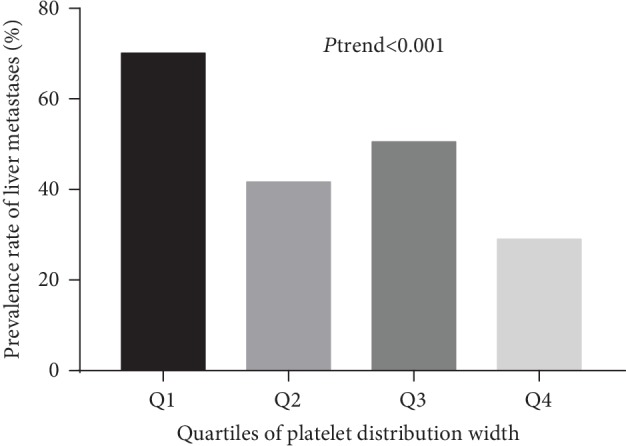
The prevalence of liver metastasis in CRC patients according to the quartiles of PDW levels.

The prevalence of liver metastasis was calculated by the quartiles of PDW levels (Figure 1). The prevalence rate of liver metastasis in quartile 1, quartile 2, quartile 3, and quartile 4 was 70.1% (82/117), 41.6% (37/89), 50.5% (54/107), and 29.0% (27/93), respectively.

The risks of liver metastasis according to PDW quartiles are analyzed and shown in Table 4. After adjusting for age, gender, BMI, smoking status, drinking status, WBC, haemoglobin, albumin, and fasting plasma glucose, the prevalence risk of liver metastasis for the highest quartile of PDW was 0.190 (0.101-0.358).

## 4. Discussion

Our study demonstrated that the CRC patients with liver metastases have lower PDW levels compared to those without metastases and PDW was independently associated with the presence of liver metastases.

The interaction of tumor cells with platelets leads to platelet activation, which in turn promotes tumor progression and metastasis [14]. Elevated platelet-derived growth factor D promotes CRC cell proliferation and invasion by upregulating the expression of Notch1 and matrix metalloproteinase 9 [15]. Moreover, platelet-derived endothelial cell growth factor levels were increased in CRC patients and were associated with poor prognosis [16, 17]. A recent study confirmed that serum platelet-derived growth factor AA is an independent predictor for CRC liver metastasis [18]. In addition, the use of low-dose aspirin could restore antitumor activity by inhibiting platelet COX-1 [19]. Our study confirmed the important role of platelet activation in CRC. Moreover, our results provide the basis for applying antiplatelet therapy in CRC patients with liver metastases.

The mechanisms underlying the association between reduced PDW and CRC liver metastases remain unclear. The interaction between platelets and tumor cells induces cell plasticity and promotes cancer metastasis by enhancing circulating tumor cell survival and extravasation [20]. PDW is an indicator of the average change in platelet volume. Platelet volume is determined both during megakaryopoiesis and thrombopoiesis. The decrease of PDW reflects the failure of heterogenic megakaryocytic maturation [21]. The thrombocytopoiesis is regulated by many factors, among which the key factor has been attributed to thrombopoietin (TPO) [22]. Elevated plasma TPO levels have been observed in cancer patients with advanced stage [23]. Recent study found that PDW was a better indicator to reflect the characteristics of activated platelets [24]. The secretory factors released by activated platelets promote the expression of cytokines, proteolytic enzymes, and chemokines within the microenvironment and accelerate cancer invasion [25]. Overexpression of platelet-derived growth factor (PDGF) was related to uncontrolled angiogenesis in CRC patients with liver metastases and was found to be a new prognostic indicator for a worse prognosis in CRC [18, 26]. In addition, overexpression of PDGF receptor is associated with advanced stage disease in stromal cells of human colon carcinomas [27]. In addition, recent studies revealed that dual antiplatelet therapy (aspirin and clopidogrel) inhibits the expression of *α*-granule-stored proteins and decreases the heterotypic interactions between platelets/leukocytes and the endothelium [28].

Our current study bears several limitations. Firstly, it was a retrospective and single center study. Secondly, the mechanisms underlying the association are needed to clarify. Thirdly, the conclusion could not be applied to other ethnic groups because the cohorts in our study were composed of Chinese patients.

In summary, compared with CRC patients without metastases, PDW is reduced in CRC patients with liver metastases. Moreover, PDW was independently associated with the presence of CRC liver metastases.

## Figures and Tables

**Table 1:** Baseline characteristics of CRC patients.

Variables	With liver metastases	Without liver metastases	*P* value
*N*	200	206	
Age (years)	59.0 ± 11.4	58.2 ± 10.0	0.423
Gender (male, %)	125 (62.5)	111 (53.9)	0.079
BMI (kg/m^2^)	23.5 ± 3.0	23.6 ± 3.4	0.936
Smoker (*n*, %)			
Ever/current (%)	54 (27.0)	47 (22.8)	0.330
Drinking status			
Ever/current (%)	45 (22.5)	49 (23.8)	0.759
FPG (mmol/L)	5.20 (4.78-5.90)	5.12 (4.73-5.46)	0.141
Albumin (g/L)	41.3 ± 6.7	43.5 ± 7.0	0.001
Haemoglobin (g/dL)	121.1 ± 24.4	128.8 ± 23.7	0.001
WBC (×10^9^/L)	7.6 ± 2.6	7.0 ± 2.6	0.012
Platelet count (×10^9^/L)	288.3 ± 102.5	271.8 ± 100.2	0.102
MPV (fL)	8.5 ± 1.0	8.3 ± 1.4	0.299
PDW (%)	16.9 ± 0.9	17.4 ± 1.0	<0.001
Primary tumor location			0.096
Colon	139 (69.5)	127 (61.7)	
Rectum	61 (30.5)	79 (38.3)	
T classification			0.006
T1+T2	21 (10.5)	42 (20.4)	
T3+T4	179 (89.5)	164 (79.6)	
Lymph node metastasis			0.006
Absent	71 (35.5)	101 (49.0)	
Present	129 (64.5)	105 (51.0)	
Differentiation			0.462
Poor	48 (72.8)	56 (27.2)	
Well/moderate	152 (24.0)	150 (72.8)	

CRC: colorectal cancer; FPG: fasting plasma glucose; WBC: white blood cell; BMI: body mass index; MPV: mean platelet volume; PDW: platelet distribution width.

**Table 2:** Platelet indices in CRC patients without liver metastases.

Variables	PLT	*P* value	MPV	*P* value	PDW	*P* value
Primary tumor location		0.005		0.905		0.605
Colon	298.1 (123.1)		8.4 (1.7)		17.4 (1.0)	
Rectum	254.5 (77.4)		8.3 (1.2)		17.3 (1.0)	
Tumor size (cm)		0.487		**0.001**		0.165
<5.0	275.2 (104.9)		8.1 (1.2)		17.3 (0.9)	
≥5.0	264.8 (89.8)		8.8 (1.7)		17.5 (1.1)	
Differentiation		0.279		0.206		0.698
Well/moderate	267.2 (94.1)		8.4 (1.4)		17.4 (1.0)	
Poor	284.2 (115.0)		8.1 (1.4)		17.3 (0.9)	
T classification		**0.035**		0.084		0.526
T1+T2	242.8 (88.8)		8.7 (1.1)		17.4 (1.1)	
T3+T4	279.3 (101.8)		8.3 (1.5)		17.3 (0.9)	
Lymph node metastasis		0.461		0.059		**0.034**
Absent	266.6 (113.8)		8.5 (1.3)		17.5 (1.0)	
Present	276.9 (85.3)		8.2 (1.5)		17.2 (0.8)	

PLT: platelet count; MPV: mean platelet volume; PDW: platelet distribution width.

**Table 3:** Platelet indices in CRC patients with liver metastases.

Variables	PLT	*P* value	MPV	*P* value	PDW	*P* value
Primary tumor location		0.251		0.614		0.778
Colon	293.8 (95.8)		8.5 (1.0)		16.9 (1.0)	
Rectum	275.8 (116.2)		8.5 (1.0)		16.9 (0.8)	
Tumor size (cm)		0.815		0.520		0.811
<5.0	286.5 (111.8)		8.5 (1.1)		16.9 (1.0)	
≥5.0	289.9 (93.9)		8.4 (1.0)		16.9 (1.0)	
Differentiation		0.476		0.378		0.600
Poor	275.5 (102.4)		8.3 (0.8)		16.8 (0.8)	
Well/moderate	290.4 (102.6)		8.5 (1.1)		16.9 (0.9)	
T classification		0.956		0.551		0.958
T1+T2	287.1 (95.9)		8.3 (1.4)		16.9 (0.7)	
T3+T4	288.5 (103.5)		8.5 (1.0)		16.9 (0.9)	
Lymph node metastasis		0.660		0.852		0.313
Absent	284.0 (84.1)		8.5 (0.9)		17.0 (0.9)	
Present	290.7 (115.5)		8.5 (1.1)		16.9 (0.9)	
Metastatic tumor size (cm)		0.815		0.520		0.811
<5.0	286.5 (111.8)		8.5 (1.1)		16.9 (0.9)	
≥5.0	289.9 (93.9)		8.4 (1.0)		16.9 (1.0)	
Metastatic tumor nodules		0.420		0.134		0.979
<2	300.4 (133.1)		8.3 (1.0)		16.9 (0.8)	
≥2	285.5 (94.1)		8.5 (1.0)		16.9 (1.0)	

PLT: platelet count; MPV: mean platelet volume; PDW: platelet distribution width.

**Table 4:** The risks of liver metastases in CRC patients according to PDW quartiles.

	Cases	Controls	OR (95% CI)	*P* value
Q1 (≤16.5%)	82	35	1 (reference)	
Q2 (16.6-17.0%)	37	52	0.289 (0.156-0.535)	<0.001
Q3 (17.1-17.7%)	54	53	0.482 (0.271-0.860)	0.013
Q4 (≥17.8%)	27	66	0.190 (0.101-0.358)	<0.001

Logistic regression analysis adjusted for age, gender, BMI, smoking status, drinking status, albumin, FPG, haemoglobin, and WBC. CI: confidence interval.

## Data Availability

The data used to support the findings of this study are available from the corresponding authors upon request.
